# A Rare Case of Steroid Cell Tumor, Not Otherwise Specified (NOS), of the Ovary in a Young Woman

**DOI:** 10.1155/2019/4375839

**Published:** 2019-07-25

**Authors:** Eek Chaw Tan, Chit Chong Khong, Kazila Bhutia

**Affiliations:** Division of Obstetrics & Gynaecology, KK Women's and Children's Hospital, Singapore

## Abstract

Steroid cell tumour is a rare sex cord-stromal tumor of the ovary. It may produce steroids and is associated with testosterone secretion which causes symptoms like hair loss, hirsutism, and oligomenorrhea/amenorrhea due to hormonal activity and virilizing properties of tumor. In this article, we reported a 27-year-old woman who presented with hirsutism, hoarseness of voice, scalp hair fall, and amenorrhea for 8 years. Clinical and diagnostic evaluation revealed a left adnexal mass and elevated serum levels of testosterone and she was diagnosed as having a Sertoli Leydig cell tumour of ovary. She underwent left salpingooophorectomy and both histopathological examination and immunohistochemistry confirmed the diagnosis. Her serum testosterone levels normalized 3 days after the surgery and her menses resumed spontaneously a few months after the operation. In addition, we reviewed the literature on the epidemiology, clinical presentations, imaging and histological findings, and the treatment options on this disease.

## 1. Introduction

Steroid cell tumour (SCT) accounts for less than 0.1% of all ovarian neoplasm [1]. It is classified into 3 categories based on the cell origin: stromal luteoma, Leydig cell tumour, and SCT not otherwise specified (NOS) [2, 3]. Steroid cell tumour may produce steroids and is associated with testosterone secretion which causes symptoms such as hair loss, hirsutism, and oligomenorrhea/amenorrhea due to hormonal activity and virilizing properties of tumor [4]. In this report, we present a case of steroid cell tumour, NOS patient who presented with amenorrhea and symptoms of virilization.

## 2. Case

A 27-year-old single virgin lady presented to our clinic with hirsutism, hoarseness of voice, scalp hair fall, and amenorrhea for the last 8 years. She had menarche at the age of 10 and menses were regular until age of 16 years. Her menses became irregular after the age of 16 and was amenorrhoeic by 19 years old. She also complained of gradual weight gain of 10 kg over the last two years to her current weight of 107 kg.

The physical examination revealed an androgenic alopecia, acanthosis nigricans, hirsutism (Ferriman Galway Score 27), and clitoromegaly. Her BMI was 43.4 kb/m^2^.

She did not have withdrawal bleed following progesterone challenge with oral medroxyprogesterone tablets for 7 days. The blood tests showed normal serum prolactin, estradiol level, free thyroxin, and thyroid stimulating hormone. However, serum total testosterone level was elevated at 22.5 nmol/L (normal range 0.4 – 2nmol/L) and serum LH and FSH were low with levels of 0.1 IU/L. In addition, both DHEA-S (dehydroepiandrosterone sulfate) and 17-hydroxy progesterone level were normal which made adrenal source unlikely.

**Figure 1:**
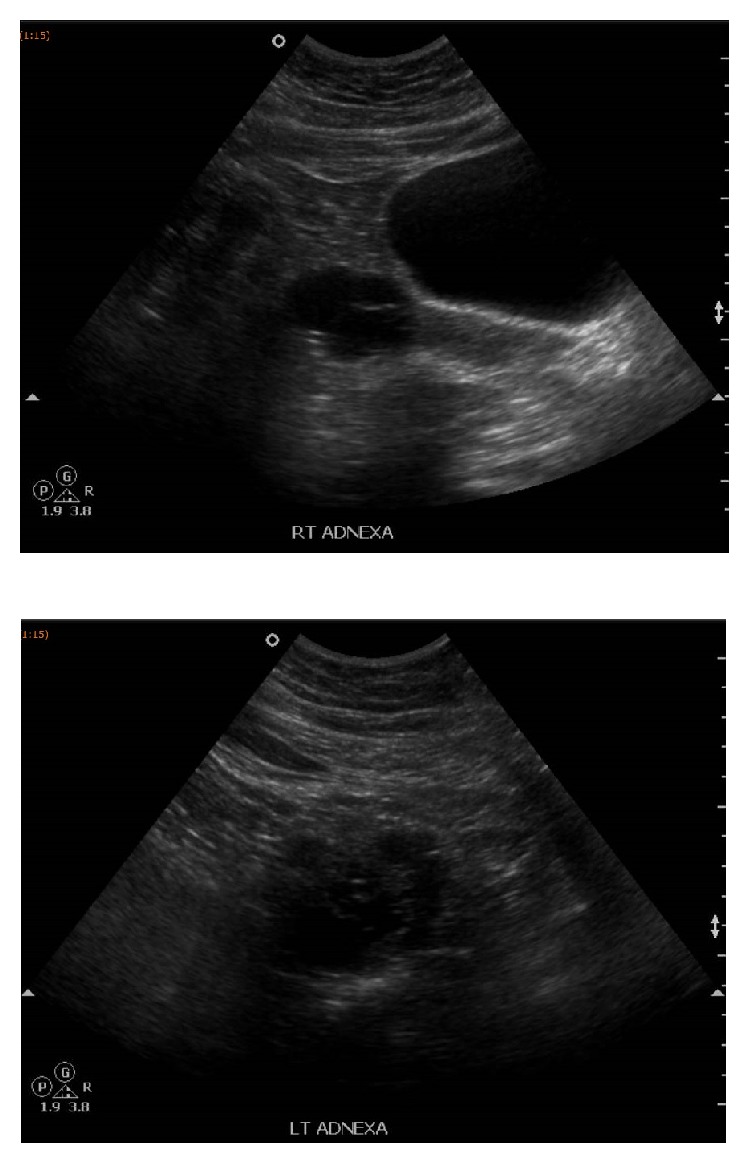
The ultrasound pelvis showed a multicystic avascular structure in the right adnexa measuring 4.5 x 4.3 x 3.3cm. Another multicystic avascular structure was seen in the left adnexa measuring 5.4 x 4.4 x 4.1cm. These could possibly represent polycystic ovaries containing multiple cysts.

**Figure 2:**
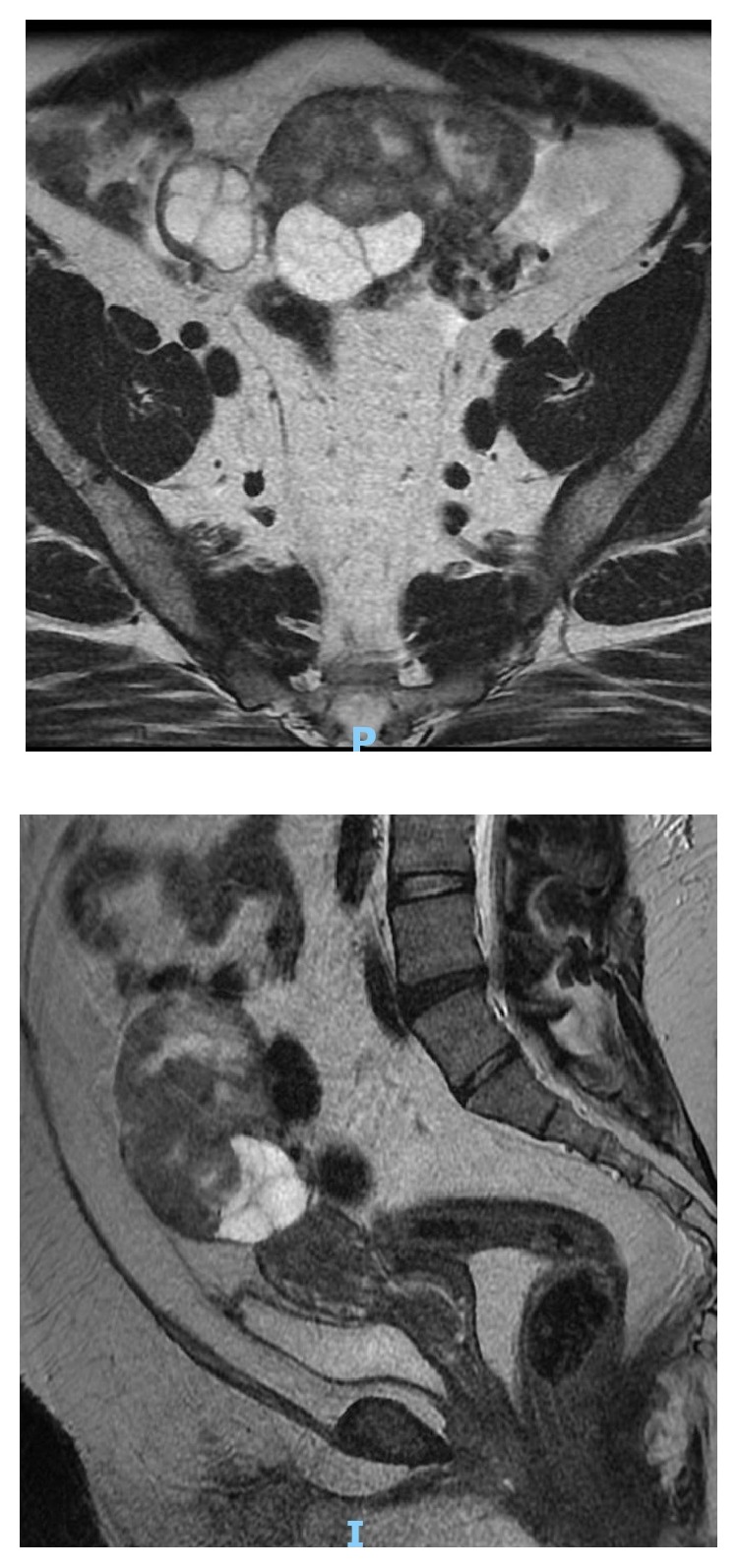
MRI abdomen and pelvis showed the presence of a large heterogeneous predominantly solid mass arising from the left ovary with cystic areas within. The solid components showed restricted diffusion and avid contrast enhancement. The mass was measured 8.4 x 6.1 x 8.9 cm. The solid components were hyperintense to muscle on T2w imaging and iso/hypointense on T1w imaging.

The transvaginal sonography showed endometrial thickness of 3 mm with polycystic ovaries containing multiple avascular cysts (Figure 1). Hence, based on initial work-up, differential diagnosis of Polycystic Ovarian Syndrome was made. MRI of abdomen and pelvis was arranged and it revealed a 8.4 x 6.1x 8.9 cm predominantly solid enhancing mass arising from the left ovary. There was no evidence of adrenal mass or abdominal or pelvic lymphadenopathy (Figure 2). Blood test for ovarian tumour markers including beta HCG, Ca 125, Ca 19-9, chorioembryonic antigen, and alpha-fetoprotein were normal.

In view of the raised serum testosterone level and MRI of pelvis findings, the initial impression was a Sertoli Leydig cell tumour of the ovary.

## 3. Treatment

**Figure 3:**
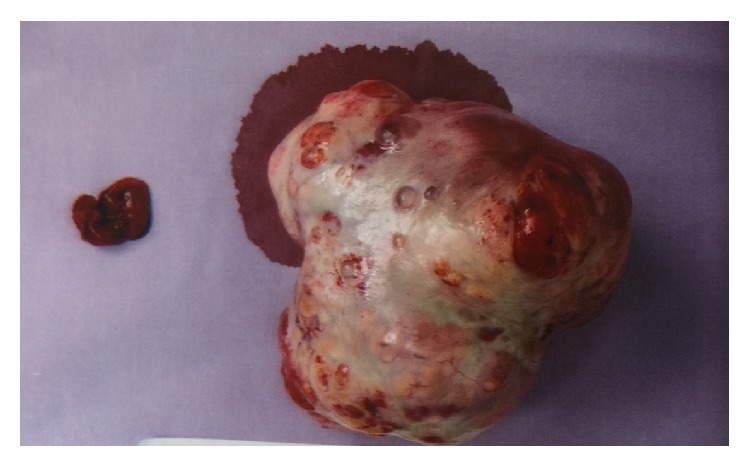
The specimen showed the left ovarian tumour and fallopian tube of the patient. The gross specimen consisted of a lobulated mass 100 x 100 x 30 mm with a defect measuring 20 mm in size externally, altogether weighing 177 grams. The mass had morcellated appearance. On cut section had solid and multicystic appearance. The solid areas had a golden yellow appearance punctuated by foci of haemorrhage. A small fleshy tan solid-cystic area was present at one end measuring 20 x 15 x 15 mm. In cytological examination, the sections of the main tumour showed a predominantly diffuse proliferation of polygonal cells with ample, pale, vacuolated cytoplasm and round central nuclei. Small areas of a more nested appearance were also seen. Foci showing cells with more eosinophilic cytoplasm were also evident, but no Reinke crystals were evident. The nuclei were central and round, with no significant atypia. Immunohistochemical stains for calretinin and alpha-inhibin were diffusely and strongly positive. CD 10 is negative. An area showing multiple cystic structures lined by bland cuboidal to columnar epithelium with focal ciliation in a fibromatous stroma was seen. No significant epithelial proliferation or atypia was seen.

The patient underwent an open laparotomy and left salpingooophorectomy. Intraoperatively, she was noted to have a 10 X 10 X 3 cm left ovarian mass (Figure 3). The uterus, bilateral fallopian tubes, and right ovary were normal. The frozen section of the left ovarian mass was reported as Sertoli Leydig cell tumor. The patient had unremarkable postoperative recovery and discharged well on the third postoperative day.

## 4. Outcome and Follow-Up

The final histology confirmed steroid cell tumour, not otherwise specified. Her serum testosterone levels normalized 3 days after the surgery and her menses had resumed spontaneously a few months after the operation. She is currently free of disease and is on regular follow-up with Gynaecological Cancer Centre.

## 5. Discussion

Steroid cell tumour was first described by Scully to contribute for less than 0.1% of all ovarian neoplasm [1, 5]. 60 % of SCT are NOS category [6] and more than 90% of the NOS are unilateral [1, 7, 8]. NOS tends to affect younger women (mean age; 43 years) [7] and 25-40 % of NOS tumors are malignant [7].

NOS are associated with androgenic changes with variable frequency ranging from 12% to over 50% and they usually stay for many years [9–12]. They generally present with virilizing symptoms such as gradually progressive hirsutism, acne, deepening of voice, temporal baldness, and amenorrhea [13]. 25% of the patients with NOS remain asymptomatic [14].

In this case, the patient was presented with irregular menses after the age of 16 years and became amenorrhoeic by the age of 19 years. She also had virilizing signs of androgenic alopecia, acanthosis nigricans, hirsutism (Ferriman Galway Score 27), and clitoromegaly.

Approximately 56-77% of patients with NOS are associated with high testosterone [13], 6-23% of the patients generally present with hyperestrogenemia [2], and 6-10% exhibit excess cortisol secretion, which may cause Cushing syndrome [15, 16].

Monteagudo et al. study had shown that the small steroid cell tumor had different echogenicity from the surrounding ovarian tissue and 66.6% of the cases had low impendence-to-flow values [17]. However, their sample size is low and further study needs to be done to prove its significance. Ultrasound feature of steroid cell tumor can be misinterpreted as polycystic ovary due to the multicystic avascular structure feature with enlarged ovary [18]. Our patient's ultrasound failed to detect the presence of an ovarian mass, which was only identified on MRI pelvis.

Histology remains the gold standard for diagnosis of NOS. The gross appearance of NOS generally is well circumscribed, solid and noncalcified with a lobulated appearance while cross section appears as yellow-orange surface with occasional cystic changes [14, 19, 20]. Microscopically, the tumor has a nested arrangement but can be organized into columns or cords like zona glomerulosa and zona fasciculate appearance. In cytological examination, polygonal or round cells with distinctive borders and central and prominent nucleoli are seen [21–24]. It is differentiated from Leydig cell tumours by absence of Reinke's crystal [25].

Immunohistochemical markers for inhibin and calretinin are sensitive markers for steroid cell tumors NOS [26]. The sensitivity of positive calretinin is 60 to 90 % whereas the sensitivity of inhibin reactivity ranges from 5 to 90 % [27, 28]. Other markers such as EMA, cytokeratin, CD 99, and S100 have been reported to be positive. HMB45, Chromogranin-A, LeuM1, AFP, carcinoembryonic antigen (CEA), and periodic acid Schiff (PAS) are other markers which have been studied [29].

The recommended treatment of steroid cell tumor, NOS is primarily surgical. For patients with benign tumour confined to one ovary and family is completed, unilateral salpingooophorectomy should be recommended. For patients with unilateral malignant tumour desiring fertility, staging laparotomy with unilateral salpingooophorectomy with preservation of contralateral ovary and uterus is a reasonable option [30]. In postmenopausal patient or bilateral tumors, hysterectomy with bilateral salpingooophorectomy can be performed [22]. For metastatic cases, debulking surgery and chemotherapy or radiotherapy are recommended [13, 22]. In this case, the patient underwent laparotomy and left salpingooophorectomy. Serum testosterone levels normalized 3 days after the surgery and her menses resumed spontaneously a few months after the operation.

## 6. Conclusion

Steroid cell tumour, NOS is a rare tumour which can be onerous to diagnose. We highlight the importance of careful laboratory and imaging investigations with involvement of the multidisciplinary team in making the diagnosis. Any young patient presenting with severe virilisation and/or menstrual problems, neoplastic etiology should always be kept in mind.

## References

[1] Young R. H., Clement P. B., Scully R. E., Mills S.-E., Carter D., Greenson J.-K., Oberman H.-A., Reuter V., Stoler M.-H. (2004). Sex-cord, stromal, steroid cell and germ cell tumours of the ovary. *Sternbergs Diagnostic Surgical Pathology*.

[2] Hayes M. C., Scully R. E. (1987). Ovarian steroid cell tumors (not otherwise specified): a clinicopathological analysis of 63 cases. *The American Journal of Surgical Pathology*.

[3] Kim Y. T., Kim S. W., Yoon B. S. (2007). An ovarian steroid cell tumor causing virilization and massive ascites. *Yonsei Medical Journal*.

[4] Revathy M., Kanchana M. P. (2018). Incidence of virilisation in sex cord stromal tumours of ovary, a 5-year experience in a tertiary care gynaecological centre. *Journal of Evolution of Medical and Dental Sciences*.

[5] Seles F. M., Revathy M., Kanchana M. P. (2018). Steroid cell tumour of the ovary: a case report with review of literature. *International Journal of Reproduction, Contraception, Obstetrics and Gynecology*.

[6] Amneus M. W., Natarajan S. (2003). Pathologic quiz case: a rare tumor of the ovary. *Archives of Pathology & Laboratory Medicine*.

[7] Outwater E. K., Wagner B. J., Mannion C., McLarney J. K., Kim B. (1998). Sex cord-stromal and steroid cell tumors of the ovary.. *RadioGraphics*.

[8] Scully R. E., Young R. H., Clement P. B. (1996). Steroid cell tumors. *Tumors of the Ovary, Mal-Developed Gonads, Fallopian Tube and Broad Ligament Washington*.

[9] Young R. H., Shully R. E., Fox H, Spain W. M. (2003). Steroid cell tumors of the ovary. *Obstetric & Gynecological Pathology*.

[10] Young R. H., Scully R. E., Kurman R. J. (1994). Sex cord-stromal, steroid cell, and other ovarian tumors with endocrine, paraendocrine, and paraneoplastic manifestations. *Blausteins Pathology of the Female Genital Tract*.

[11] Powell J. L., Dulaney D. P., Shiro B. C. (2000). Androgen- secreting steroid cell tumor of the ovary. *Southern Medical Journal*.

[12] Paraskevas M. (1989). Schully Re: Hilus cell tumor of the ovary. *International Journal of Gynecological Pathology*.

[13] Sood N., Desai K., Chindris A., Lewis J., Dinh T. A. (2017). Symptomatic ovarian steroid cell tumor not otherwise specified in a post-menopausal woman. *Rare Tumors*.

[14] Wang P.-H., Chao H.-T., Lee R.-C. (1998). Steroid cell tumors of the ovary: clinical, ultrasonic, and MRI diagnosis—a case report. *European Journal of Radiology*.

[15] Sawathiparnich P., Sitthinamsuwan P., Sanpakit K., Laohapensang M., Chuangsuwanich T. (2009). Cushing’s syndrome caused by an ACTH-producing ovarian steroid cell tumor, NOS, in a prepubertal girl. *Endocrine Journal*.

[16] Yuan M., Qiu M., Zhu M. (2014). Symptomatic cushing syndrome and hyperandrogenemia revealing steroid cell ovarian neoplasm with late intra-abdominal metastasis. *BMC Endocrine Disorders*.

[17] Monteagudo A., Heller D., Husami N., Levine R. U., McCaffrey R., Timor-Tritsch I. E. (1997). Ovarian steroid cell tumors: sonographic characteristics. *Ultrasound in Obstetrics & Gynecology*.

[18] Alves P., Sá I., Brito M., Carnide C., Moutinho O. (2019). An early diagnosis of an ovarian steroid cell tumor not otherwise specified in a woman. *Case Reports in Obstetrics and Gynecology*.

[19] Tsai H., Chen S., Wei H., Chen G. (2009). Hypothyroidism and hyperlipidemia with a virilizing ovarian steroid cell tumor, not otherwise specified. *Gynecological Endocrinology*.

[20] Boyraz G., Selcuk I., Yusifli Z., Usubutun A., Gunalp S. (2013). Steroid cell tumor of the ovary in an adolescent: a rare case report. *Case Reports in Medicine*.

[21] Reedy M. B., Richards W. E., Ueland F. (1999). Ovarian steroid cell tumors, not otherwise specified: a case report and literature review. *Gynecologic Oncology*.

[22] Li K., Zhu F., Xiong J., Liu F. (2014). A rare occurrence of a malignant ovarian steroid cell tumor not otherwise specified: a case report and literature review. *Oncology Letters*.

[23] Yuan M., Qiu M., Zhu M. (2014). Symptomatic Cushing syndrome and hyperandrogenemia revealing steroid cell ovarian neoplasm with late intra-abdominal metastasis. *BMC Endocrine Disord*.

[24] Jiang W., Tao X., Fang F., Zhang S., Xu C. (2013). Benign and malignant ovarian steroid cell tumors, not otherwise specified: case studies, comparison, and review of the literature. *Journal of Ovarian Research*.

[25] Zhang X., Lü B. (2011). Ovarian steroid cell tumor, not otherwisespecified (nos). *International Journal of Gynecological Pathology*.

[26] Zhao C., Vinh T. N., McManus K., Dabbs D., Barner R., Vang R. (2009). Identification of the most sensitive and robust immunohistochemical markers in different categories of ovarian sex cord-stromal tumors. *The American Journal of Surgical Pathology*.

[27] Deavers M. T., Malpica A., Ordonez N. G., Silva E. G. (2003). Ovarian steroid cell tumors: an immunohistochemical study including a comparison of calretinin with inhibin. *International Journal of Gynecological Pathology*.

[28] Movahedi-Lankarani S., Kurman R. J. (2002). Calretinin, a more sensitive but less specific marker than *α*-inhibin for ovarian sex cord-stromal neoplasms: an immunohistochemical study of 215 cases. *The American Journal of Surgical Pathology*.

[29] Jones M. W., Harri R., Dabbs D. J., Carter G. J. (2010). Immunohistochemical profile of steroid cell tumor of the Ovary: a study of 14 cases and a review of the literature. *International Journal of Gynecological Pathology*.

[30] Boyraz G., Selcuk I., Yusifli Z., Usubutun A., Gunalp S. (2013). Steroid cell tumor of the ovary in an adolescent: a rare case report. *Case Reports in Medicine*.

